# Ensemble perception requires attention

**DOI:** 10.3758/s13414-025-03111-9

**Published:** 2025-06-18

**Authors:** Ruth Kimchi, Shahar Sabary

**Affiliations:** 1 School of Psychological Sciences, University of Haifa, Haifa, Israel; 2 Institute of Information Processing and Decision Making, University of Haifa, Haifa, Israel

**Keywords:** Ensemble perception, Attention, Unattended processing, Implicit processing, Automatic processing

## Abstract

The question of whether ensemble perception can take place without attention is unresolved. We examined this issue in four experiments, using an inattention paradigm that provides an on-line, indirect measure of processing of unattended stimuli. Participants performed an attention-demanding change-detection task on a small matrix presented on a background of task-irrelevant ensemble consisting of circles of different size (Experiment 1) or oriented lines (Experiments 2–4). Independently of any change in the matrix, the ensemble mean changed or stayed the same between successive displays on each trial. We hypothesized that if ensemble mean is extracted under inattention, changes in the ensemble mean would produce congruency effects on the speed or accuracy of performance in the matrix change judgments, such that performance is faster or more accurate on congruent than incongruent trials. The results showed that changes in the ensemble mean size or mean orientation produced no congruency effects on performance of the target change-detection task. Also, participants could not report, when probed with surprise questions, whether or not the ensemble mean changed. When participants attended to the ensemble, their accuracy of explicit reports about a change were significantly above chance. These results are seen to suggest that ensemble perception requires attention. The differences between the present study and previous ones, concerning the conditions and definition of unattended and their implication for understanding the relation between ensemble perception and attention, are discussed.

## Introduction

Facing a vast amount of information and limited resources, the perceptual system exploits environmental statistical regularities and redundancy and rapidly forms ensemble perception – a summary representation of a groups of similar objects.

Ensemble perception has been reported for low-level information such as average direction and speed of motion (e.g., Watamaniuk, Sekuler, & Williams, 1989), average orientation (e.g., Alvarez & Oliva, 2009; Parkes et al., 2001), average spatial position (Alvarez & Oliva, 2008), average hue (Maule & Franklin, 2015; Webster, Kay, & Webster, 2014), and average size (e.g., Ariely, 2001; Chong & Treisman, 2003), as well as for high-level information such as average facial expression and gender (Haberman & Whitney, 2007, 2009), average facial identity (Neumann, Schweinberger, & Burton, 2013), and average gaze direction (Sweeny & Whitney, 2014). The ability to extract the statistical property of multiple visual items is not limited to the mean representation. Ensemble variance and ensemble range were also documented (e.g., Haberman et al., 2015; Khayat & Hochstein, 2018) (for reviews, see Corbett, Utochkin, & Hochstein, 2023; Whitney & Yamanashi, 2018.)

An unresolved issue is whether ensemble perception can occur without attention. A number of studies have addressed this question, and the picture that emerges is far from being consistent. On the one hand, several studies have suggested that ensemble perception can take place without attention or with minimal attention (e.g., Alvarez & Oliva, 2008, 2009; Bronfman, Brezis, Jacobson, & Usher, 2014; Chen, Zhuang, Wang, Ren, & Abrams, 2021; Chong & Treisman, 2005b; Joo, Shin, Chong, & Blake, 2009; Liu & Ji, 2024; Oriet & Brand, 2013). For example, Alvarez and Oliva (2008, 2009) reported that participants were equally accurate in estimating the final centroid of both attended and unattended sets of moving dots and were able to detect changes in the average orientation of the background. Bronfman et al. (2014) showed that participants could report the color diversity of letters outside the focus of attention with no cost to the performance of their primary letter task (see also Ward, Bear, & Scholl, 2016), and Liu and Ji (2024) found that extracting mean facial emotion was not affected by attentional load. Studies with patients with unilateral spatial neglect (USN) are also seen to suggest that unattended elements can contribute to ensemble perception (e.g., Pavlovskaya, Soroker, Bonneh, & Hochstein, 2015). On the other hand, a number of studies have shown that ensemble perception requires attention (Huang, 2015; Jackson-Nielsen, Cohen, & Pitts, 2017; Lukashevich et al., 2025; McNair et al., 2017). For example, Jackson-Nielsen et al. (2017), using a modified inattention paradigm, showed that a majority of the participants were inattentionally blind to color diversity, size diversity, and mean size, and McNair et al. (2017) found that mean emotional expression of a set of faces was affected by attentional blink. Lukashevich et al. (2025) conducted an event-related potential (ERP) study and found no evidence for the marker vMMN (visual mismatch negativity), which is elicited by unattended change discrimination, in response to a change in the mean orientation of an unattended ensemble.

The inconsistency of the findings may be partly due to the use of the terms “processing without attention,” “automatic processing,” and “unconscious processing” interchangeably, which although could be related, are distinguished. Methodology also seems to play an important part, as noted by Chen et al. (2021). Thus, several studies suggesting that ensemble perception is attention free have used dual-task paradigms (e.g., Alvarez & Oliva, 2008, 2009; Bronfman et al., 2014; Liu & Ji, 2024). It is possible, however, that in these paradigms participants allocated some attentional resources to the secondary ensemble task, thus preventing deriving clear conclusions regarding the attentional demands of ensemble processing. The studies suggesting that attention is necessary for ensemble perception have used different paradigms, such as inattention paradigms (e.g., Jackson-Nielsen et al., 2017), which are free from this particular concern but are subject to the possibility that apparent failures of ensemble perception attributed to absence of attention may be actually due to failures of memory, a recognized problem with Mack and Rock’s (1998) original inattention paradigm (see Wolfe, 1999). Chen and colleagues (2021) used a method that was less prone to these two weaknesses. Participants were required to attend to one of two ensembles of lines and to indicate whether a pre-specified target category (vertical, horizontal, or oblique orientation) was present. The results showed that the unattended, to-be-ignored ensemble influenced performance on the attended ensemble only when it matched the target category, suggesting that whether or not ensemble perception can take place outside focal attention may be dependent on attentional control setting.

In the present study we examined whether ensemble perception can take place without attention using an inattention paradigm, which also does not have the shortcomings of some of the previous studies mentioned above. This paradigm provides indirect online measures for the processing of unattended stimuli by engaging participants in an attention-demanding change-detection task at the center of the display and examining the influence of the unattended task-irrelevant background on the target task (e.g., Driver et al., 2001; Kimchi & Peterson, 2008; Kimchi & Razpurker-Apfeld, 2004; Russell & Driver, 2005). In each trial, participants were briefly presented with two successive displays, each comprising a small target matrix surrounded by circles of different size or by orientated lines. The target matrix can slightly change between two successive displays or remain the same. Independently of any change in the matrix, the mean of the unattended background ensemble (mean size in Experiment 1 and mean orientation in Experiments 2–4) changed or remained the same. The individual ensemble elements always changed between successive displays regardless of whether or not the mean changed, to control for the possibility that a change could be detected from a change in the individual elements per se. We hypothesized that if means were extracted for the unattended background ensembles, then congruency effects would emerge, such that responses to *same* targets would be faster and/or more accurate when the ensemble mean stayed the same than when it changed, and responses to *different* targets would be faster and/or more accurate when the ensemble mean changed than when it stayed the same. After the last experimental trial, observers were probed with surprise questions about the immediately preceding background ensemble.

To foreshadow the results, changes in the unattended ensemble mean produced no congruency effects on performance of the target change-detection task, and participants could not report, in response to the surprise questions, whether or not the ensemble mean changed. When participants attended to the background ensemble, while ignoring the central matrix, their accuracy of explicit reports about a change in the mean were significantly above chance. These results are seen to suggest that ensemble perception requires attention.

### General methods

#### Participants

All participants were students at the University of Haifa and were paid or granted a course credit for participation. All participants provided informed consent to a protocol approved by the Ethics Committee of the University of Haifa and were informed of the option of exiting the experiment at any time. All participants had normal vision and none participated in more than one experiment. The sample size for the inattention experiments and for the focused attention experiments was based on previously reported sample sizes in studies using the inattention paradigm (Kimchi & Razpurker-Apfeld, 2004; Rashal et al., 2017; Russell & Driver, 2005).

#### Apparatus

Stimuli were generated using Matlab R2014a and Psychophysics Toolbox (http://psychtoolbox.org). The experiments were designed and stimulus presentation was controlled by E-Prime 3 software (Psychology Software Tools, Pittsburgh, PA, USA). All stimuli were presented on an LCD BenQ monitor (24-in., 100-Hz refresh rate, 1,920 × 1,080 resolution). Participants provided responses using a response-box (Psychology software tools, model 200 A) and a computer keyboard. Participants viewed the screen through a circular aperture (16 cm in diameter) of a matte black cardboard sheet. A chin rest was used to set the viewing distance to approximately 57 cm from the monitor. The testing room was dimly lit.

#### Stimuli

Each display consisted of a central target matrix surrounded by a background ensemble. The central target matrix in Experiments 1–3 contained 12 black (RGB: 0,0,0) and 13 white (RGB: 255,225,225) small solid squares, 0.2° each, randomly located in a 5 × 5 matrix subtending 1.0° × 1.0°. The central target stayed the same between two successive displays on half of the trials and changed on the other half. The change was made by switching the location of one small black square with that of a white one. The central target in Experiment 4 contained 11 vertical and five horizontal black lines equally spaced and randomly located in a 4 × 4 matrix (see details in the method section of Experiment 4).

**Fig. 1:**
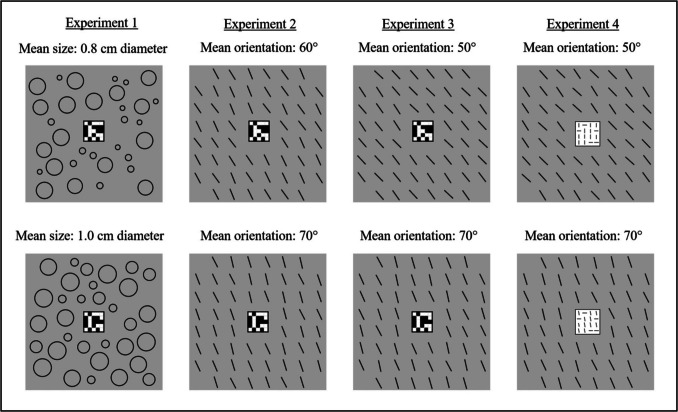
Examples of the displays presented in Experiments 1–4. Each display consisted of a central target matrix surrounded by an ensemble comprising outline circles of different sizes in Experiment 1 and lines of different orientations in Experiments 2–4. There were two possible means for each ensemble (see text for details). Stimuli are a bit darker in the figure for illustration purposes only

The ensemble elements (outline circles of different sizes in Experiment 1 and lines of different orientations in Experiments 2–4) were medium gray (RGB: 120,120,120) presented on a light gray background (RGB: 192,192,192), located in a 9.4° × 9.4° square (Experiment 1) and 8° × 8° square (Experiments 2–4). In each experiment, there were two possible means of the background ensemble (two mean sizes in Experiment 1 and two mean orientations in Experiments 2–4). For each mean there were two sets of elements (a and b), which varied in individual elements but had the same mean. Examples of the stimuli in the four experiments are presented in Fig. 1.

#### Design and procedure

In each experiment participants completed 160 experimental trials in two blocks of 80 trials each, preceded by one practice block of 16 trials. A 2 (target: same, different) × 2 (ensemble mean: same, different) within-subjects design was used. All the combinations of target and ensemble mean were randomized within blocks, with each combination occurring on an equal number of trials. Half of the trials were same-target trials, and half were different-target trials. Independently of whether the target changed or remained the same on each trial, the ensemble mean changed or remained the same. The individual ensemble elements (or subgroup of the elements) always changed between successive displays, independently of a change in the ensemble mean, to control for the possibility of detecting a change in the ensemble from a local change in the size (Experiment 1) or orientation (Experiments 2–4) of just a few (or even one) elements.

### Inattention experiments (Experiments 1a–3a and 4)

These experiments were designed to investigate whether ensemble perception can take place without attention.

**Fig. 2:**
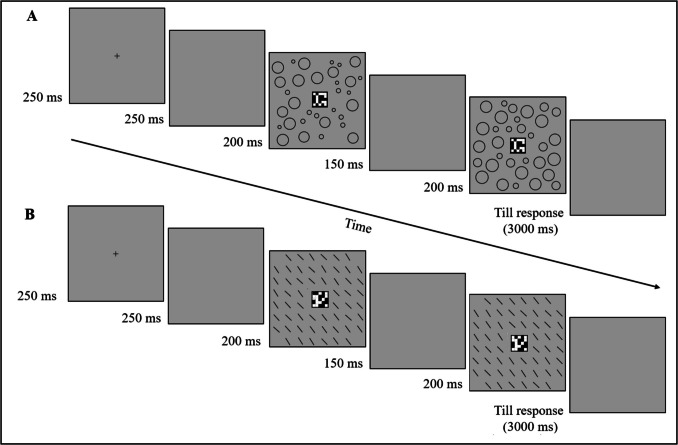
Sequence of events in a trial. The illustration depicts two examples of trials: (**A**) a same-target (central matrix is unchanged) different-ensemble (mean size of the circles changes) trial (from Experiment 1), and (**B**) a different-target (central matrix changes) same-ensemble (mean orientation of the lines does not change) trial (from Experiment 3)

The sequence of events in a trial is presented in Fig. 2. Each trial started with a fixation mark (0.3° × 0.3° black cross, RGB: 0,0,0) presented at the center of the screen for 250 ms. After a 250-ms interval, the first display appeared for 200 ms followed by a 150-ms interval, and then the second display appeared for 200 ms. At this point, the participants had to decide, as rapidly and as accurately as possible, whether the two successive central matrices were the same or different by pressing one of two response keys. Reaction time (RT) was measured from the appearance of the second display until a response was made. An auditory tone provided immediate feedback after an incorrect response or when 3,000 ms had elapsed with no response. The inter-trial interval was 1,000 ms. Immediately after participants responded to the last experimental trial, they were asked two forced-choice questions regarding the background ensemble in the preceding trial. The first question asked was, “Did you see anything else on the screen other than the central matrix?” The participants had to choose between “Yes” and “No” by pressing one of two response keys. The second question asked was tailored to each experiment, depending on the background ensemble elements. In Experiment 1a the second question asked was, “Was there a difference in the average size of the circles that appeared in the background between the last two pictures (guess if you do not know)?” and in Experiments 2a–3a and 4 it was, “Was there a difference in the average orientation of the lines that appeared in the background between the last two pictures (guess if you do not know)?” The participants had to choose between “There was a difference” and “There was no difference” by pressing one of two response keys. Subjects who responded negatively to the first question were nevertheless encouraged to answer the second question.

### Focused attention experiments (Experiments 1b–3b)

In these experiments we examined whether changes in the means of the background ensembles could be perceived when attention was allocated to them.

The stimuli, design, and procedure were the same as in the inattention experiments, except that participants were instructed to focus on the surrounding elements while ignoring the central matrix, and to indicate, immediately after the second display in a trial disappeared, as accurately as possible, whether the average size of the circles (Experiment 1b) or the average orientation of the lines (Experiments 2b and 3b) in the two successive displays in a trial is the same or different, by pressing one of two response keys. No forced-choice questions appeared after subjects responded to the last experimental trial.

### Data analysis

#### Online performance on the matrix task in the inattention experiments

All RT summaries and analyses are based on participants’ mean RTs for correct responses. Trials in which RT was shorter than 200 ms or longer than 2,000 ms were excluded from the analyses. Repeated-measures analyses of variance (ANOVAs) and one-tailed paired t-tests were used to analyze the RT and accuracy data. All ANOVAs and t-tests were calculated using SAS (version 9.4).

When null effects were theoretically important, i.e., inferring that attention may be necessary for ensemble perception to occur, we also evaluated evidence in favor of the null hypothesis by computing the Bayes factor (BF10) in a Bayesian paired t-test, using JASP statistical software (www.jasp-stats.org) and a Cauchy prior centered on zero (scale = 0.707).

#### Perception of changes in ensemble means in the focused attention experiments

We calculated two measures: percent correct and sensitivity for detecting a change, i.e., a signal-detection *d’.* The latter was calculated for each participant as z(HR)-z(FA), where HR (hit rate) is the proportion of correctly responded ensemble-different trials and FA (false alarm) is the proportion of incorrectly responded ensemble-same trials. We report the average *d’* across participants.

## Experiment 1

### Method

#### Participants

Twenty-three (19 females, one left-handed, age range: 19–36 years, M = 23.7 years) participated in Experiment 1a, and 18 (15 females, one left-handed, age range: 20–33 years, M = 25.17 years) participated in Experiment 1b.

#### Stimuli

The ensemble elements were 32 outline circles (with contour of 2 pixels in width) containing four different diameter sizes, each diameter appeared eight times. The circles were randomly placed within the square area (see Fig. 1a), with a minimum distance of 0.2 cm between each other and a minimum distance of 0.2 cm between the most central circles and the central target matrix. This was made in order to keep the circles’ density in the displays as similar as possible. There were two possible mean sizes of the background ensembles: diameter of 0.8 cm or diameter of 1.0 cm. For each mean diameter there were two sets of circles. For the mean diameter 0.8, set 0.8a included circles of diameters of 0.4, 0.5, 1.1, and 1.2 cm, and set 0.8b included circles of diameters of 0.5, 0.6, 0.9, and 1.2 cm. For the mean diameter of 1.0 cm, the circles in set 0.1a had diameters of 0.6, 0.9, 1.2, and 1.3 cm, and the circle in set 1.0b had diameters of 0.7, 0.9, 1.1, and 1.3 cm. The mean size of the background ensemble either remained the same between two successive displays or changed. Some of the circles in a display always changed their size from the first to the second display regardless of whether or not their mean size changed. Thus, when the mean size of 0.8 cm remained the same, the first display consisted of set 0.8a and the second of set 0.8b, or vice versa, such that half of the circles changed their size from the first to the second display. Similarly, when the mean size of 1.0 remained the same, set 1.0a was paired with set 1.0b. When the mean size changed, set 0.8a was paired with set 1.0a and set 0.8b was paired with set 1.0b.

## Results

### Experiment 1a

One participant was excluded from the analyses as accuracy on the target task was at chance.

#### Online performance on the matrix task

**Fig. 3:**
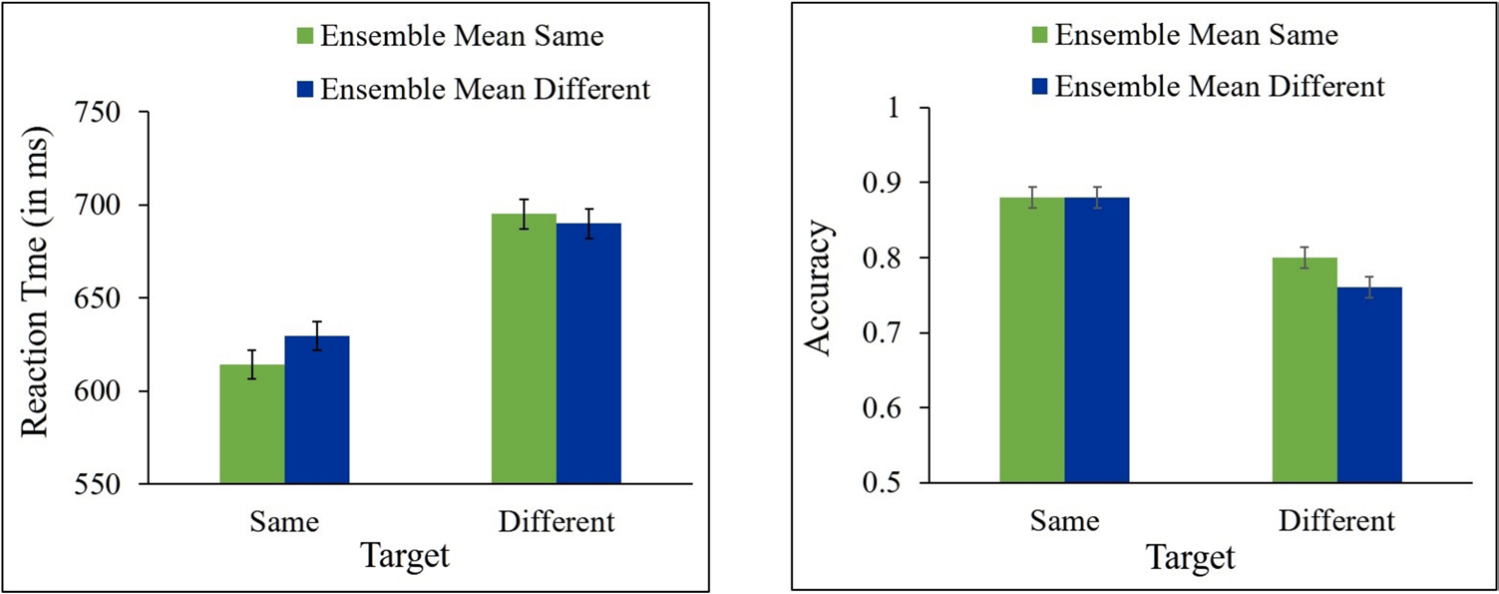
Results of Experiment 1a. Mean correct reaction time (**left panel**) and accuracy (**right panel**) for same and different targets as a function of ensemble mean (same, different). Error bars represent within-subjects SEM

In all RT analyses, trials in which responses to the target were incorrect (17.07% of trials) were excluded from the analyses. In addition, trials in which RT was shorter than 200 ms or longer than 2,000 ms were also excluded (2.1% of all trials). Mean correct RT and mean accuracy for same and different responses as a function of ensemble change are presented in Fig. 3.

A 2 (target: same, different) × 2 (ensemble mean size: same, different) repeated-measures ANOVAs were performed on the RT and accuracy data. The analyses showed a significant effect of target [*F*(1, 21) = 29.93, *p* < 0.0001, ƞ_p_^2^ = 0.59, *F*(1, 21) = 10.26, *p* = 0.0043, ƞ_p_^2^ = 0.33, for RT and accuracy, respectively], indicating that participants were faster and more accurate in “same” than in “different” responses. This effect, which was found also in the next experiments, is reminiscent of the fast-same effect (e.g., Farell, 1985). The main effect of ensemble was significant for accuracy [*F*(1, 21) = 4.46, *p* = 0.0469, ƞ_p_^2^ = 0.18] but not for RT, *F* < 1. No significant interaction between target and ensemble was found for RT [*F*(1, 21) = 1.74, *p* = 0.2012, ƞ_p_^2^ = 0.07], nor for accuracy [*F*(1, 21) = 1.80, *p* = 0.1937, ƞ_p_^2^ = 0.08]. As can be seen in Fig. 3, responses to different targets were not faster nor more accurate when ensemble mean size changed across the two displays than when it was unchanged [*t*(21) = 0.44, *p* = 0.3321, *t*(21) = −1.98, *p* = 0.9696, for RT and accuracy, respectively]. Responses to same targets were not significantly faster nor more accurate when ensemble mean size stayed the same than when it changed [*t*(21) = 1.63, *p* = 0.0587, *t*(21) = 0.29, *p* = 0.3888, for RT and accuracy, respectively]. Bayesian paired t-tests show that the evidence provides anecdotal support in RT (BF_10_ = 0.322) and strong support in accuracy (BF_10_ = 0.0849) for the null hypothesis for the different targets, and substantial support in accuracy (BF_10_ = 0.281) for the null hypothesis and anecdotal support in RT (BF_10_ = 1.306) for the alternative hypothesis for the same targets.

Taken together, these results show that changes in the ensemble mean size did not produce congruency effects on performance in the target change-detection task.

#### Responses to the surprise questions

Percentages of participants who responded correctly to each surprise question are presented in Table 1. The percentage of participants (73%) who reported seeing something else besides the central target was significantly above chance, χ^2^(1) = 4.5455, *p* = 0.033. However, the percentage of participants (55%) who correctly reported whether or not there was a difference in the mean size of the circles that appeared in the background on the preceding trial was not different from chance, χ^2^(1) = 0.1818, *p* = 0.6698.

**Table 1:** Percentage of participants who responded correctly to each forced-choice surprise question in Experiments 1a–4

	**Forced-choice question**	
	Something	Change in background mean
Experiment 1a	73% (16/22)	55% (12/22)
Experiment 2a	65% (15/23)	43% (10/23)
Experiment 3a	64% (14/22)	45% (10/22)
Experiment 4	61% (14/23)	52% (12/23)

These results show that participants reported seeing the background ensemble, but they failed to report whether or not the background ensemble mean had changed during the trial.

### Experiment 1b

Participants correctly reported whether or not the mean size of the circles had changed during the preceding trial on 71% of the trials, which is significantly above chance [*t*(17) = 7.12, *p* < 0.0001, Cohen’s *d* = 1.68], and there was no difference between performance in different-mean (71%) and same-mean (72%) trials [*t*(17) = −0.43, *p* = 0.6629]. The average *dʹ* was 1.25 (SD = 0.841), indicating moderate performance.

## Discussion

The lack of congruency effects arising from changes in the background ensemble mean size (Experiment 1a) suggests that the mean size of a group of circles varying in size could not be extracted under conditions that satisfy criteria for inattention as proposed by Mack and Rock (1998; see also, Moore, Grosjean, & Lleras, 2003). The target change-detection task was sufficiently demanding to absorb attention (mean accuracy 82.93%), the ensemble mean size was irrelevant to the task, and participants performed poorly in reporting a change in it; these considerations strongly suggest that the background ensemble was unattended. When attention was allocated to the ensemble (Experiment 1b), participants’ accuracy in reporting whether or not the mean size of the circles had changed during the preceding trial was significantly above chance, with moderate sensitivity to changes in the ensemble means.

Thus, the results of the present experiment are seen to suggest that ensemble perception of size requires attention.

It should be noted that although we attempted to reduce confound between the mean size of the circles and their density as much as possible, such confound may have been present, and could have potentially affected the interpretation of the results, had unattended processing been found. In the absence of any indication of processing of changes in the unattended ensemble, it is reasonable to take the present results as suggesting that ensemble perception of size may not be carried out without attention, regardless of any possible effect of ensemble density.

In the next experiment we examined whether ensemble perception of orientation can take place without attention.

## Experiment 2

### Method

#### Participants

Twenty-three individuals (19 females, three left-handed, age range: 20–48 years, M = 24.52 years) participated in Experiment 2a and 16 (12 females, two left-handed, age range: 19–29 years, M = 22.50 years) participated in Experiment 2b.

#### Stimuli

The background ensemble elements were 56 lines arranged in an 8 × 8 imaginary grid (8 cm × 8 cm) excluding eight lines – four central lines and one in each corner (see Fig. 1b). Lines positions were jittered by 0.02 cm to avoid elements homogeneity. The lines subtended 0.7º in length and 2 pixels in width. There were eight different orientations (each orientation appeared seven times), and two possible mean orientations: 60º or 70º (90º is vertical). For each mean orientation there were two sets of lines. For the mean orientation 60º, set 60a included orientations ranged from 52º to 68º in steps of 2º, and set 60b included orientations ranged from 53º to 67º in steps of 2º. For the mean orientation of 70º, the orientations in set 70a ranged from 62º to 78º in steps of 2º, and in set 70b the orientations ranged from 63º to 77º in steps of 2º. The mean orientation of the ensemble of lines either remained the same between two successive displays or changed. All of the ensemble lines always changed their orientation from the first to the second display regardless of whether or not their mean orientation changed. Thus, when the mean orientation of 60º remained the same, the first display consisted of set 60a and the second of set 60b, or vice versa, and similarly, when the mean orientation of 70º remained the same, set 70a was paired with set 70b.When the mean orientation changed, set 60a was paired with set 70b and set 60b was paired with set 70a.

## Results

### Experiment 2a

#### Online performance on the matrix task

**Fig. 4:**
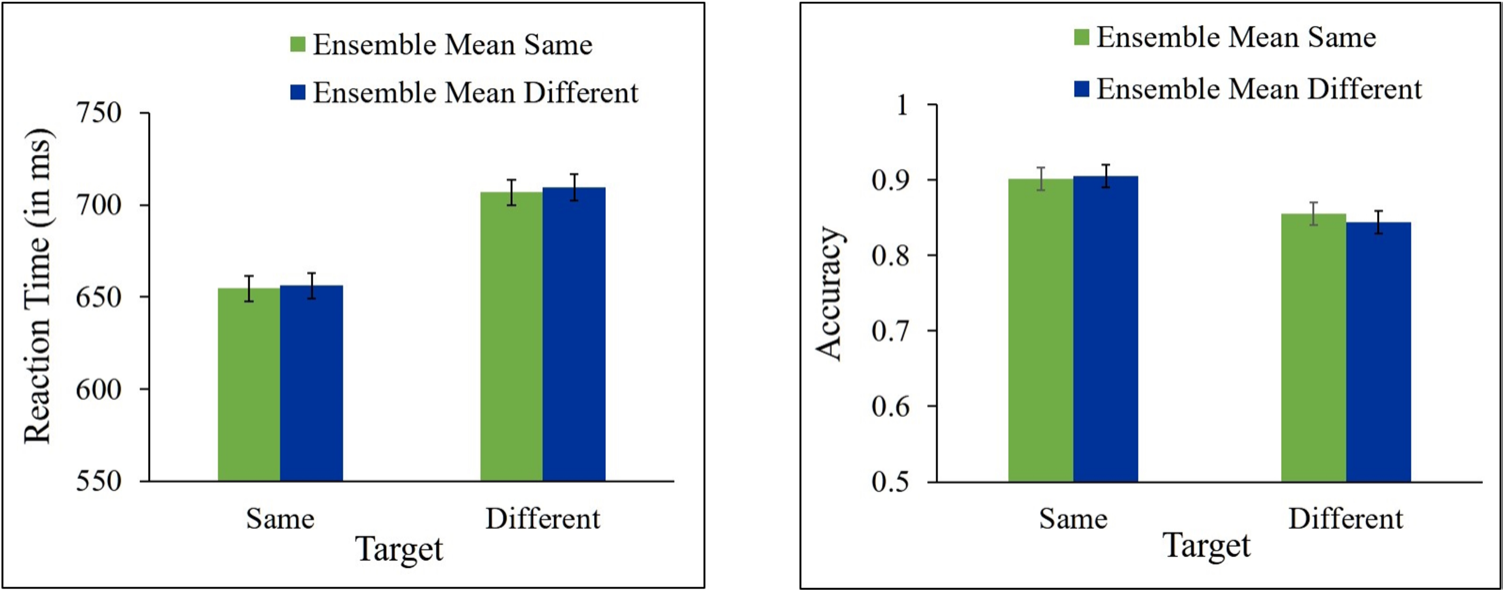
Results of Experiment 2a. Mean correct reaction time (**left panel**) and accuracy (**right panel**) for same and different targets as a function of ensemble mean (same, different). Error bars represent within-subjects SEM

In all RT analyses, trials in which responses to the target were incorrect (12.45% of trials) were excluded from the analyses. In addition, trials in which RT was shorter than 200 ms or longer than 2,000 ms were also excluded (0.43% of all trials). Mean correct RTs and mean accuracy for same and different responses as a function of ensemble change are presented in Fig. 4.

A 2 (target: same, different) × 2 (ensemble mean orientation: same, different) repeated-measures ANOVA showed a significant effect of target [*F*(1, 22) = 41.33, *p* < 0.0001, ƞ_p_^2^ = 0.65, *F*(1, 22) = 4.39, *p* = 0.0479, ƞ_p_^2^ = 0.17, for RT and accuracy, respectively], indicating that participants were faster and more accurate in “same” than in “different” responses. The main effect of ensemble was not significant, *F*s < 1, for RT and accuracy. No significant interaction between target and ensemble was found, *F*s < 1, for RT and accuracy. As can be seen in Fig. 4, responses to different targets were not faster nor more accurate when ensemble mean orientation changed across the two displays than when it was unchanged [*t*(22) = −0.24, *p* = 0.5951, *t*(22) = −0.78, *p* = 0.7779, for RT and accuracy, respectively], and responses to same targets were not faster nor more accurate when ensemble mean orientation stayed the same than when it changed [*t*(22) = 0.15, *p* = 0.4402, *t*(22) = −0.15, *p* = 0.5602, for RT and accuracy, respectively]. Bayesian paired t-tests show that the evidence provides substantial support in RT (BF_10_ = 0.184) and in accuracy (BF_10_ = 0.134) for the null hypothesis for the different targets, and substantial support in RT (BF_10_ = 0.246) and in accuracy (BF_10_ = 0.196) for the null hypothesis for the same targets.

These results show that changes in the background ensemble mean orientation produced no congruency effects on performance in the target change-detection task.

#### Responses to the surprise questions

Percentages of participants who responded correctly to each surprise question are presented in Table 1. The percentage of participants (65%) who reported seeing something else besides the central target was not significantly above chance, χ^2^(1) = 2.1304, *p* = 0.1444, and the percentage of participants (43%) who correctly reported a change in the mean orientation of the lines was also no different from chance, χ^2^(1) = 0.3913, *p* = 0.5316.

These results show that participants performed at chance in reporting seeing something else besides the central target and in reporting whether or not the background ensemble mean had changed during the trial.

### Experiment 2b

Participants correctly reported whether or not the mean orientation of the lines had changed during the preceding trial on 68% of the trials, which is significantly above chance [*t* (15) = 6.44, *p* < 0.0001, Cohen’s *d* = 1.61], and there was no significant difference between performance in different-mean (69%) and same-mean (68%) trials [*t* (15) = 0.24, *p* = 0.4061]. The average *dʹ* was 1.04 (SD = 0.693), indicating quite a moderate performance.

## Discussion

The lack of congruency effect arising from changes in the background ensemble mean orientation (Experiment 2a) suggests that the mean orientation of a group of lines varying in orientation could not be extracted under conditions that, like in Experiment 1a, satisfy criteria for inattention (Mack & Rock, 1998). The target change-detection task was sufficiently demanding to absorb attention (mean accuracy 87.55%), the ensemble mean orientation was irrelevant to the task, and participants performed at chance in reporting seeing something besides the central matrix and in reporting whether the mean orientation had changed during the preceding trial. When attention was allocated to the background ensemble (Experiment 2b), participants’ accuracy in reporting whether or not the mean orientation of the lines had changed during the preceding trial was above chance, with a moderate sensitivity to mean changes.

Taken together, the results of the present experiment are seen to suggest that ensemble perception of orientation cannot take place without attention.

We take the findings that changes in the background ensemble mean size and mean orientation produced no congruency effects on the target change-detection task (Experiments 1a and 2a) as indicating that estimates of mean size and mean orientation cannot be extracted without attention. One may argue, however, that the lack of congruency effects was a result of low discriminability of the means: Even when attention was allocated to the background ensemble, performance at reporting a change in the mean size (Experiments 1b) or in the mean orientation (Experiment 2b) between two successive displays was only moderate, average *dʹ* = 1.25 (71% accuracy) and *d*ʹ = 1.04 (68% accuracy), for mean size and mean orientation, respectively.

To address this alternative account, we conducted the next experiment that was similar to Experiment 2, except that the discriminability of the ensemble means orientation was higher. If the lack of congruency effects in Experiment 2a is accounted for by low discriminability of the ensemble means, then congruency effects are expected to emerge when the discriminability of the means is higher. If, however, the lack of congruency effects is due to the inability to extract the mean without attention, then we expect to replicate the results of Experiment 2a, providing further evidence that ensemble perception of orientation requires attention.

## Experiment 3

### Method

#### Participants

Twenty-three individuals (15 females, six left-handed, age range: 19–41 years, M = 25.65 years) participated in Experiment 3a, and 12 (ten females, all right-handed, age range: 19–44 years, M = 28.50) participated in Experiment 3b.

#### Stimuli

Stimuli were the same as in Experiment 2, except that the two possible mean orientations of the ensemble were 50º and 70º (Fig. 1). As in Experiment 2, for each mean orientation there were two sets of lines, each included eight different orientations. For the mean orientation 50º, set 50a included lines of orientations ranged from 42º to 58º with steps of 2º, and set 50b included lines that had orientations ranged from 43º to 57º with steps of 2º. For the mean orientation of 70º, the lines had the same orientations as in Experiment 2. In half of the same-mean trials the mean orientation was of 50º (set 50a was paired with set 50b), and in the other half it was 70º (set 70a was paired with set 70b), and in half of the different-mean trials, set 50a was paired with set 70b and in the other half set 50b was paired with set 70a.

## Results

### Experiment 3a

One participant was excluded from the analyses as accuracy on the target task was at chance.

#### Online performance on the matrix task

**Fig. 5:**
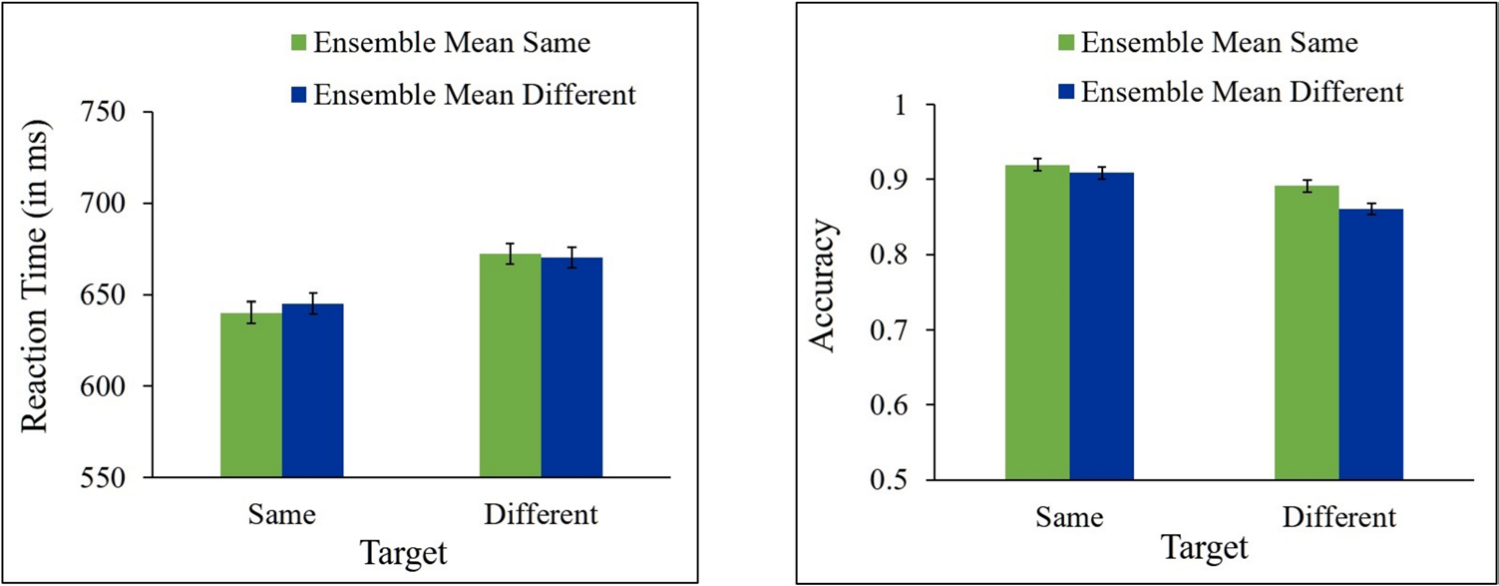
Results of Experiment 3a. Mean correct reaction time (**left panel**) and accuracy (**right panel**) for same and different targets as a function of ensemble mean orientation (same, different). Error bars represent within-subjects SEM

In all RT analyses, trials in which responses to the target were incorrect (10.63% of trials) were excluded from the analysis. In addition, trials in which RT was shorter than 200 ms or longer than 2,000 ms were also excluded (0.63% of all trials). Mean correct RTs and mean accuracy for same and different targets as a function of ensemble mean change are presented in Fig. 5.

A 2 (target: same, different) × 2 (ensemble mean orientation: same, different) repeated measures ANOVA was performed on the RT and accuracy data. The analysis showed a significant effect of target for RT [*F*(1, 21) = 5.24, *p* = 0.0325, ƞ_p_^2^ = 0.2], but not for accuracy [*F*(1, 21) = 3.43, *p* = 0.0780, ƞ_p_^2^ = 0.14], indicating that participants were faster in "same" than in "different" responses. The main effect of ensemble was not significant for RT, *F* < 1, nor for accuracy [*F*(1, 21) = 3.12, *p* = 0.0920, ƞ_p_^2^ = 0.12]. No significant interaction between target and ensemble was found for RT, *F* < 1, nor for accuracy [*F*(1, 21) = 1.27, *p* = 0.2724, ƞ_p_^2^ = 0.06]. As can be seen in Fig. 5, responses to different targets were not faster nor more accurate when background ensemble mean orientation changed across the two displays than when it was unchanged [*t*(21) = 0.2, *p* = 0.4207, *t*(21) = −2.12, *p* = 0.9771, for RT and accuracy, respectively], and responses to same targets were not faster nor more accurate when background ensemble mean orientation stayed the same than when it changed [*t*(21) = 0.51, *p* = 0.3085, *t*(21) = 0.72, *p* = 0.2397, for RT and accuracy, respectively]. Bayesian paired t-tests show that the evidence provides substantial support in RT (BF_10_ = 0.262) and strong support in accuracy (BF_10_ = 0.0815) for the null hypothesis for the different targets, and anecdotal support in RT (BF_10_ = 0.342) and in accuracy (BF_10_ = 0.422) for the null hypothesis for the same targets.

These results show that changes in the background ensemble mean orientation did not produce congruency effects on performance in the target change-detection task.

#### Responses to the surprise questions

Percentages of participants who responded correctly to each surprise question are presented in Table 1. The percentage of participants (64%) who reported seeing something else besides the central target was not significantly above chance, χ^2^(1) = 1.6364, *p* = 0.2008, and the percentage of participants (45%) who correctly reported a change in the mean orientation of the lines was also no different from chance, χ^2^(1) = 0.1818, *p* = 0.6698.

These results replicated the results of Experiment 2, showing that participants performed at chance in reporting seeing something else besides the central target and in reporting whether or not the background ensemble mean had changed during the trial.

### Experiment 3b

Participants correctly reported whether or not the mean orientation of the lines had changed during the preceding trial on 92% of the trials, which is significantly above chance [*t* (11) = 31.68, *p* < 0.0001, Cohen’s *d* = 9.14] and there was no significant difference between performance in different-mean (91%) and same-mean (92%) trials [*t* (11) = −0.59, *p* = 0.7151]. Average *dʹ* was 2.89 (SD = 0.691), indicating high sensitivity to a change.

## Discussion

The present results clearly show that increasing the discriminability of the background ensemble mean orientation resulted in higher performance in detecting a change in the mean orientation when the ensemble was attended (Experiment 3b), but it had no effect on the results in the unattended condition (Experiment 3a). The results of Experiment 3a replicated the results of Experiment 2a. Participants were engaged in the target change-detection task (mean accuracy 89.38%), they performed at chance in reporting whether the mean orientation had changed during the preceding trial, and changes in the ensemble mean orientation produced no congruency effects on performance in the target change-detection task. These findings rule out the alternative account that the lack of congruency effects observed in Experiment 2 is a result of low discriminability of the ensemble means.

A question may arise whether the absence of congruency effects would be observed, were the differences between ensemble means larger. The difference of 20º between ensemble means in our experiment yielded high sensitivity to change when ensembles were attended, but the sensitivity could be lower when the same ensembles are unattended. It is worth pointing out, however, that our results show that increasing the discriminability of the means twice as much (from a difference of 10º to 20º) had no effect whatsoever on the results under inattention, replicating the absence of congruency effects.

Alvarez and Oliva (2009) showed that participants were able to detect a large change of 45º in mean orientation of background ensembles while performing an attention-demanding motion tracking task. Yet, monitoring changes in the background ensembles was a secondary task, and therefore, as we noted earlier, some attention was allocated to the background ensembles. Indeed, Alvarez and Oliva emphasized that they examined ensemble perception under reduced attention, not under inattention. Hence, further research is required to examine whether the absence of congruency effects that we observed generalizes to very large differences between means of unattended ensembles, as it has the potential of delineating the conditions under which unattended change detection can occur, if at all.

Taken together, the results of Experiments 1–3 clearly show that the unattended background ensemble had no influence on the target task, suggesting that no ensemble perception (of size or of orientation) occurred in the absence of attention.

On the face of it, these results appear to disagree with the results of Chen et al. (2021), who also used an implicit measure of unattended processing and found that the unattended ensemble influenced performance on the attended ensemble. Importantly, however, the influence of the unattended ensemble took place only when the properties of the unattended ensemble matched the properties of the target. This clearly was not the case in our Experiments 1–3, in which the dimension of change in the background ensemble was dissimilar to the dimension of change in the target.

In the next experiment we examined whether similarity between the domains of change of the background and the target would influence performance such that changes in the background ensemble would produce congruency effects on the target change detection. To this end we used a central target matrix composed of mostly vertical lines that could stay the same or slightly change their orientation between successive displays. The background ensemble was the same as in Experiment 3, and the ensemble mean orientation could stay the same or change independently of a change in the target orientation.

## Experiment 4

### Method

#### Participants

Twenty-three (16 females, three left-handed, age range: 19–30 years, M = 23.39 years) participated in Experiment 4.

#### Stimuli

Background ensemble stimuli were the same as in Experiment 3. The central target contained 11 vertical (90° or 80°) and five horizontal (180°) black (RGB: 0,0,0) lines, each of 0.25° in length and 1 pixel in width, equally spaced and randomly located in a 4 × 4 matrix subtending 1.5° × 1.5° (see Fig. 1). The central target stayed the same between two successive displays on half of the trials and changed on the other half. The change was made by slightly changing the orientation of the vertical lines. In target-same trials, the vertical lines on two successive displays in a trial were either 90° on both displays or 80° on both displays. In target-different trials, the vertical lines changed between the two successive displays from 90° to 80° or vice versa. The spatial arrangement of the lines was always the same in successive displays in a trial, but varied across trials.

## Results

### Online performance on the matrix task

In all RT analyses, trials in which responses to the target were incorrect (11.96% of trials) were excluded from the analyses. In addition, trials in which RT was shorter than 200 ms or longer than 2,000 ms were also excluded (0.98% of all trials).

**Fig. 6:**
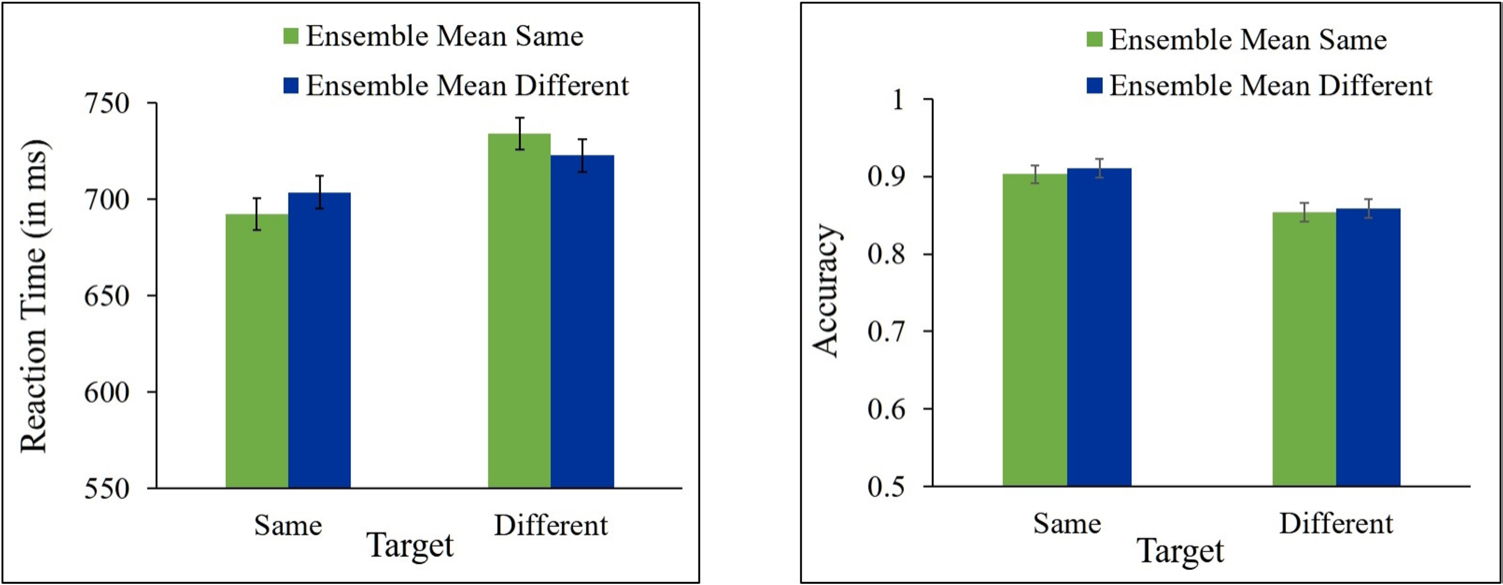
Results of Experiment 4. Mean correct reaction time (**left panel**) and accuracy (**right panel**) for same and different targets as a function of ensemble mean orientation (same, different). Error bars represent within-subjects SEM

Mean correct RTs and mean accuracy for same and different targets as a function of background ensemble change are presented in Fig. 6. A 2 (target: same, different) × 2 (background ensemble mean orientation: same, different) repeated-measures ANOVA showed a significant effect of target for RT [*F*(1, 22) = 7.66, *p* = 0.0112, ƞ_p_^2^ = 0.26] but not for accuracy [*F*(1, 22) = 3.82, *p* = 0.0636, ƞ_p_^2^ = 0.14], indicating that participants were faster in "same" than in "different responses". The main effect of background ensemble was not significant for RT nor for accuracy, *Fs* < 1. There was no significant interaction between target and background ensemble mean orientation [*F*(1, 22) = 1.83, *p* = 0.1898, ƞ_p_^2^ = 0.07, *F* < 1, for RT and accuracy, respectively]. Responses to different targets were not significantly faster nor more accurate when background ensemble mean orientation changed across the two displays than when it was unchanged [*t*(22) = 1.03, *p* = 0.1563, *t*(22) = 0.30, *p* = 0.3835, for RT and accuracy, respectively], and responses to same targets were not significantly faster nor more accurate when background mean orientation stayed the same than when it changed [*t*(22) = 1.04, *p* = 0.1542, *t*(22) = −0.80, *p* = 0.7837, for RT and accuracy, respectively]. Bayesian paired t-tests show that the evidence provides anecdotal support in RT (BF_10_ = 0.587) and substantial support in accuracy (BF_10_ = 0.279) for the null hypothesis for the different targets, and anecdotal support in RT (BF_10_ = 0.593) and substantial support in accuracy (BF_10_ = 0.132) for the null hypothesis for the same targets.

### Responses to the surprise questions

Percentages of participants who responded correctly to each surprise question are presented in Table 1. The percentage of participants (61%) who reported seeing something else besides the central target was not significantly above chance, χ^2^(1) = 1.0870, *p* = 0. 2971, and the percentage of participants (52%) who correctly reported a change in the mean orientation of the lines was also no different from chance, χ^2^(1) = 0.0435, *p* = 0.8348.

These results, similarly to the results of the previous experiments, show that participants performed at chance in reporting seeing something else besides the central target and in reporting whether or not the background ensemble mean had changed during the trial.

## Discussion

The results of Experiment 4 show that manipulating the similarity of the domain of change between the unattended ensemble and the central target did not influence performance in the target change task. As in Experiment 3a, participants were engaged in the demanding target change-detection task (mean accuracy 88.04%), they performed at chance in reporting whether the mean orientation had changed during the preceding trial, and changes in the background ensemble mean orientation produced no congruency effects on performance in the target change-detection task, suggesting that ensemble perception did not occur for the unattended stimuli.

These results still appear to be inconsistent with the ones obtained by Chen et al. (2021), in which the unattended ensemble influenced performance, but only when the unattended ensemble matched the target category. For example, if the target category was vertical, unattended vertical ensemble influenced performance, whereas unattended horizontal or oblique ensembles had no influence on performance. Possibly, in our experiment, although the dimension of change in the background ensemble and the target was similar (i.e., a change in orientation), the change in the ensemble mean orientation from 50° to 70° (or vice versa), did not match exactly the change in the target vertical orientation from 90° to 80° (or vice versa), and therefore no significant influence of the unattended ensemble was observed.

Another possible explanation for the discrepancy in the results concerns the unattended stimuli. In Chen et al.’s experiment, participants attended one of two ensemble stimuli while the other was positioned outside the focus of attention and was task-irrelevant so there was no incentive for the participants to partially divide their attention between the two ensembles. However, the two ensembles were potentially relevant and participants were aware of them because on different trials one or the other ensemble could be cued. In our experiment, on the other hand, not only the target task was highly attention demanding, but through the entire experiment participants performed only the target task and were not asked to do anything regarding the background ensemble (except for the surprise questions at the end of the experiment), so the background ensemble was absolutely irrelevant, participants had no reason to deploy attention to the background ensemble and they were largely unaware of it. Thus, the background ensemble in our experiment was possibly unattended in a stricter sense comparing to the irrelevant ensemble in Chen et al.’s experiment, presumably resulting in lack of influence of the unattended ensemble. We return to this point in the *General discussion*.

## General discussion

This study examined whether ensemble perception of size and orientation can take place without attention, using an inattention paradigm that provides an online, indirect measure of processing of unattended stimuli. In all experiments, participants performed a demanding change-detection task on a small matrix presented on a background of task-irrelevant ensemble, the mean of which (mean size or mean orientation) changed or stayed the same between successive displays, independently of changes in the target. Participants in all experiments were faster in “same” responses than in “different” responses, reminiscent of the fast-same effect (e.g., Farell, 1985). Changes in the unattended ensemble mean size (Experiment 1a) or mean orientation (Experiments 2a-4) produced no congruency effects on performance in the target-change task, even when the dimensions of change in the background ensemble and in the target were similar (Experiment 4). When attended to the ensemble, participants’ reports of a change were significantly above chance. We showed that the absence of congruency effects cannot be accounted for by low discriminability of the ensemble means. Increasing the discriminability of the orientation means (Experiment 3) resulted in high accuracy (92%) of reporting a change in the mean orientation when the ensemble was attended (Experiment 3b), but it had no effect on the results in the unattended condition (Experiment 3a), demonstrating no congruency effects.

Taken together, the results of the present experiments are seen to suggest that ensemble perception, at least of size and orientation, requires attention.

A concern for any attempt to examine processing without attention is whether or not unattended conditions are indeed achieved. We employed conditions that satisfy Mack and Rock’s (1998) criteria for inattention. Consequently, the procedure we have used in the present experiments appears to be stricter than other procedures used to studying unattended processing in general and unattended ensemble perception in particular. We have already noted that in dual-task experiments, the secondary ensemble stimuli are considered unattended (e.g., Alvarez & Oliva, 2008; Bronfman et al., 2014), but the participants are aware of their presence and know that they are task-relevant and therefore are likely to deploy some attention to the secondary ensemble task. As noted in the discussion of Experiment 4, even when participants attend one of two ensemble stimuli while the other is positioned outside the focus of attention and is task-irrelevant (e.g., Chen et al., 2021), the two ensembles are potentially relevant and participants are aware of them because each ensemble could be potentially cued. In our experiments, participants performed *only* the target task through the entire experiment and they were unaware of the properties of the task-irrelevant background ensemble or any changes in it, suggesting that the background ensembles in our experiments were unattended in the strictest sense. Our results clearly show that under these conditions, task-irrelevant ensembles had no influence on the target performance. Note that in Lukashevich et al.’s (2025) ERP study the ensembles were also completely task irrelevant and unattended, and the results showed no indication that a change in the ensemble mean was registered. Taken together, these neural results and our behavioral results suggest that ensemble perception requires attention.

In light of the differences in defining unattended stimuli, or to be more exact, in the requirements for being unattended, between the various procedures used to examine the question whether or not ensemble perception requires attention, it is perhaps not surprising that the answer may depend on the procedure used to examine the question.

It should be further noted that there is one more difference between our experiments and previous experiments examining the relationship between ensemble perception and attention, which is not related to the inattention conditions per se. Rather, it relates to the task requirements. In most, if not all previous studies, ensemble perception was explicitly required, either for the secondary task (e.g., Alvarez & Oliva, 2008, 2009; Bronfman et al., 2014; Liu & Ji, 2024) or for the relevant task (e.g., Chen et al., 2021). No explicit ensemble task was present in our experiments, certainly not in Experiments 1–3, but not even in Experiment 4, in which participants were required to indicate whether the orientation of the vertical lines in two successive displays was same or different, because this task could be accomplished by inspecting the orientation of a single vertical line.

Is it possible that we observed no congruency effects because ensemble perception cannot be implicit, rather than due to absence of attention? Several studies have claimed to provide evidence for implicit ensemble perception. In these studies, no instruction to perform an ensemble task is given, but the ensemble stimuli are nevertheless attended (e.g., Chetverikov, Campana, & Kristjansson, 2016; Hansmann-Roth et al., 2021; Khayat & Hochstein, 2018; Khayat, Pavlovskaya, & Hochstein, 2024; Maule et al., 2014). For example, Khayat and Hochstein (2018) found evidence for implicit perception of mean of a set of elements. They presented participants with a rapid serial visual presentation (RSVP) sequence of elements varying in size, orientation or brightness, and then tested their memory only for membership by indicating which of two test stimuli was present in the preceding RSVP sequence. Results showed that participants tended to select the test stimulus that was closer to the set mean, even when it was never presented, suggesting that the mean was implicitly encoded. Clearly, however, participants attended the RSVP sequence in order of perform the requested task. Similarly, Hansmann-Roth et al. (2021) requested participants to search for an odd-one-out target among heterogeneous distractors and found encoding of mean, variance, and shape of the distractor distributions, suggesting implicit ensemble perception. However, performing the visual search likely required attentional suppression of the distractors. In our study, not only there was no explicit ensemble task, but the ensembles were unattended. This was also the case in Lukashevich et al.’s (2025) ERP study. Hence, our behavioral results and the neural results of Lukashevich et al. suggest that no implicit ensemble perception takes place when ensembles are not attended.

Related to this discussion is the notion of automaticity. The inattention conditions employed in our experiments seem to fit characteristics of automaticity. Although there is no single definition of automaticity, automatic processes are often characterized as operating fast, unintentionally, without attention and outside of awareness (e.g., Bargh, 1997; Carr, 1992; Hasher & Zacks, 1979; Moors & De Houwer, 2006; Posner, 1978). Thus, it may be argued that the inattention paradigm that we used actually examined whether ensemble perception is automatic (see, Tzelgov, 2000, for a similar argument regarding the inattention paradigm). If we follow a broad definition of automaticity, which means no attention, no intention and no awareness, our results are seen to suggest that ensemble perception is not automatic. These results appear to disagree with the view, held by many researchers of ensemble perception, that the process of ensemble perception is automatic (e.g., Alvarez, 2011; Corbett et al., 2023). However, processes are often considered automatic when just one or two of the above mentioned characteristics hold.

In the study of ensemble perception, automaticity sometimes refers to processing under distributed mode of attention (e.g., Alvarez, 2011; Baek & Chong, 2020; Chong & Treisman, 2005a). According to this view, ensemble perception occurs automatically when attention is distributed over multiple similar items (e.g., Treisman, 2006). It should be noted, however, that research examining whether distributed mode of attention is involved in ensemble perception has yielded mixed results (see Corbett et al., 2023, for a review).

Automaticity refers, quite often, to processing without intention, namely, to implicit ensemble perception (e.g., Corbett & Oriet, 2011; Khayat & Hochstein, 2018; Khayat et al., 2024). The present findings, however, that show no implicit ensemble perception in the absence of attention, call for a finer theoretical distinction between implicit versus automatic ensemble perception.

Before concluding we should discuss a possible limitation of our study. We have concluded, based on the absence of congruency effects on the target performance, that ensemble perception requires attention. One could argue, however, as did one of our reviewers, that unlike presence of congruency effects that clearly indicates processing without attention, absence of congruency effects may be due to reasons other than necessity of attention.

One reason is conditions that do not support ensemble perception even when the ensembles are attended. We demonstrated, however, that participants’ accuracy of correctly reporting a change in means was above chance when the ensembles were attended (Experiments 1b–3b). A second reason may be a weak same/different representation of ensemble means under inattention, which is insufficient to drive congruency effects. This is not very likely given the finding that accuracy and sensitivity to change can get quite high (92% and *d*ʹ = 2.89, respectively), and yet no congruency effects were observed under inattention (Experiment 3).

A third possibility is that for whatever reason the same/different representation of the background ensemble did not interact with the same/different representation of the target. This seems quite unlikely in light of a number of previous studies that used the same paradigm, with results that show congruency effects for some processes but not for others, depending on the complexity of the process under study. For example, Kimchi and Razpurker-Apfeld (2004) found that grouping by color similarity into rows/column produced congruency effects on the target change-detection performance but grouping by color similarity into a shape did not, suggesting that the former, a simple grouping, can take place without attention, whereas the latter, more complex grouping, requires attention. Similarly, Rashal et al. (2017) found presence of congruency effects for basic grouping by proximity, suggesting that it occurs under inattention, but absence of congruency effects for grouping by shape similarity, suggesting the necessity of attention as this grouping requires scrutiny to be accomplished.

Thus, notwithstanding the required caution, interpreting the finding of absence of congruency effects, which was consistent in four experiments, as suggesting that ensemble perception requires attention seems quite reasonable.

To summarize, we examined whether attention is necessary for ensemble perception to take place, by examining the influence of changes in unattended, completely task-irrelevant ensembles, on performance in a highly attention-demanding change-detection task. Under these strict inattention conditions our results show no influence of the unattended ensembles on the target task, suggesting that ensemble perception does not occur in the absence of attention. This conclusion regarding the dependence of ensemble perception on attention, challenges the view that the function of ensemble perception is the early, automatic, attention-independent formation of summary representations that can serve to guide attention.

## Data Availability

Data from all experiments are available via the Open Science Framework repository (https://osf.io/p78yu/).

## Code availability

Code for experimental programs and data analyses are available upon request.

## Declarations

### Open practices statement

The data of all experiments are available via the Open Science Framework at https://osf.io/p78yu/. Program code is available upon request.
